# A stochastic model for the probability of malaria extinction by mass drug administration

**DOI:** 10.1186/s12936-017-2010-x

**Published:** 2017-09-18

**Authors:** Peter Pemberton-Ross, Nakul Chitnis, Emilie Pothin, Thomas A. Smith

**Affiliations:** 1 Department of Epidemiology and Public Health, Swiss Tropical and Public Health Institute, 4051 Basel, Switzerland; 2 University of Basel, Petersplatz 1, Basel, Switzerland

**Keywords:** Stochastic extinction, Malaria, Elimination, Mathematical model

## Abstract

**Background:**

Mass drug administration (MDA) has been proposed as an intervention to achieve local extinction of malaria. Although its effect on the reproduction number is short lived, extinction may subsequently occur in a small population due to stochastic fluctuations. This paper examines how the probability of stochastic extinction depends on population size, MDA coverage and the reproduction number under control, *R*_*c*_. A simple compartmental model is developed which is used to compute the probability of extinction using probability generating functions. The expected time to extinction in small populations after MDA for various scenarios in this model is calculated analytically.

**Results:**

The results indicate that mass drug administration (Firstly, *R*_*c*_ must be sustained at *R*_*c*_ < 1.2 to avoid the rapid re-establishment of infections in the population. Secondly, the MDA must produce effective cure rates of >95% to have a non-negligible probability of successful elimination. Stochastic fluctuations only significantly affect the probability of extinction in populations of about 1000 individuals or less. The expected time to extinction via stochastic fluctuation is less than 10 years only in populations less than about 150 individuals. Clustering of secondary infections and of MDA distribution both contribute positively to the potential probability of success, indicating that MDA would most effectively be administered at the household level.

**Conclusions:**

There are very limited circumstances in which MDA will lead to local malaria elimination with a substantial probability.

**Electronic supplementary material:**

The online version of this article (doi:10.1186/s12936-017-2010-x) contains supplementary material, which is available to authorized users.

## Background

Many malaria control programmes have achieved high coverage of vector control interventions but the infection remains endemic. Mass drug administration (MDA) is one additional intervention that has been proposed as a key to eliminating the residual transmission. MDA, the time-limited distribution of drugs to a population irrespective of infection status, is a key component of many parasite elimination programs, in particular against lymphatic filariasis [1] and onchocerciasis [2]. However while MDA substantially reduces the prevalence of malaria parasitaemia in the short-term, only a few studies conducted on small islands [3], in highland settings [4], or against outbreaks [5] have shown sustained impact and in high transmission settings, there is a rapid return to pre-intervention levels of endemicity [6]. Consequently the World Health Organization recommends the use of MDA for the elimination of malaria only in areas approaching interruption of transmission where there is good access to treatment, effective implementation of vector control and surveillance, and a minimal risk of re-introduction of infection [7].

In general, the threshold condition for persistence of a pathogen is that the basic reproduction number, should be greater than unity [8], so elimination programs should exploit the most efficient strategy to reduce the reproduction number. However MDA changes the reproduction number only for the short period when there is a prophylactic effect of treatment. Mathematical models of the impact of MDA against malaria transmission agree that without some other sustained change, such as improved vector control, the effects of MDA on prevalence are likely to be transient [9]. The possibility of elimination of malaria by MDA on islands arises because extinction is a stochastic event that can occur in a small pathogen population even if the prevailing reproduction number under control is greater than unity.

To understand the basic drivers of stochastic extinction induced by MDA, only a very simple model is needed, since phenomena such as vector biology, acquired immunity, and pathogenesis are only marginally relevant. This paper uses such a model to provide a simple guide to the requirements for an MDA program to achieve elimination of any species of human malaria.

The probability of spontaneous elimination in compartmental models of infectious disease, and the expected time to its occurrence, have been extensively analysed, mostly using SIR (Susceptible-Infected-Recovered) models for pathogens that induce sterile immunity. The expressions obtained show a dependence on the population size, the initial number of infectives in the population, and the reproduction number under control. In SIR models there is a critical community size below which the infection cannot persist [10], because the population of susceptibles is exhausted. With malaria, this critical size has never been estimated and is certainly very small because the parasite can continually reinfect the same host.

As MDA affects the number of infectives but not the population size or reproduction number under control, a first approximation of the probability of elimination after MDA would be obtained using these expressions where the number of infectives is scaled by the MDA coverage.

Compartmental models require the assumption of “well-mixedness” within each compartment, whereby each individual in a compartment has an equal effect on the disease state as all other individuals in the same compartment. This notion of “exchangeability” of individuals within the same compartment implies a notion of homogeneity in the transmission dynamics. Strong heterogeneity of transmission potential or intervention coverage may, however, lead to quite different behaviours. If areas of higher intervention coverage tend to overlap with (or are effectively targeted to) areas of above-average transmission, then larger falls in cases result and the probability of elimination increases accordingly.

In the classical Susceptible-Infected-Susceptible (SIS) compartmental model, homogeneous “well-mixedness” in the entire population implies that each case is likely to produce the same number of secondary cases in the population. In such a model the effect of an MDA is easily included by instantaneously reducing the number of infected individuals by a given proportion. What is usually termed the “coverage” of the MDA intervention is the fraction of the population who receive some (or all) of the drug doses. This does not necessarily correspond to the fraction of extant infections in the population which are treated. Even if questions of treatment compliance and drug resistance are neglected, operational constraints often mean that administration of the drug to the population is clustered spatially in a way which correlates with clustering of cases. An example of this would be a health facility catchment area containing a rural area around a village, where MDA coverage is highest close to the village centre where the force of infection is lower than in the surroundings.

## Methods

A human population of size *N* is considered, with malaria infection initially at an endemic equilibrium (with $I^{ *}$ infected and infectious individuals), and a discrete-time SIS model [11] is used to relate $I^{ *}$ to the reproduction number under control *R*_*c*_ (defined as the expected number of secondary infections generated per primary infection at the prevailing coverage of control measures).

In this model

$$I_{t+1} = \frac{\beta}{N} I_t \left(N-I_t\right) + \gamma I_t, $$

and

$$I^{ *} = \frac{\beta + \gamma - 1}{\beta /N},$$

where *β*, the transmission parameter, is the expected number of new infections per infectious individual at the next time step, and *γ* is the proportion of infections that persist in the infectious state to the next time step, and:

$$R_{c} = \frac{\beta }{1 - \gamma }.$$

In the case where *I* << *N*, it is assumed that there exists a constant probability distribution $\left\{ {q_{k} } \right\}$ that each infection has the same probability of producing *k* secondary infections at the next timepoint. It can be shown [12] that

$$R_{c} = \mathop \sum \limits_{k = 0}^{\infty } kq_{k}.$$

Defining the probability generating function $g\left( x \right) = \sum\nolimits_{i = 0}^{n} {q_{i} x^{i} }$ and letting *z*_*n*_ denote the probability that the infection chain arising from each individual infection will be extinct after *n* timesteps, it can then be shown that *z*_*n*_ satisfies a recurrence relation

$$z_{n} = q_{0} + \sum\limits_{k = 1}^{\infty } {q_{k} (z_{n - 1} )^{k} } = g(z_{n - 1} ).$$

Thus for a given distribution of secondary cases the recurrence relation can be used to calculate the probability of elimination.

In the most models, it is assumed that the number of secondary cases have a Poisson distribution around the mean *R*_*c*_, i.e.

$$q_{k} = \frac{{e^{{ - kR_{c} }} }}{k!}R_{c}^{k},$$

which corresponds to a homogeneous, unclustered propagation. More generally however, the clustering of the secondary cases is incorporated by using an overdispersed distribution such as the negative binomial, and letting

$$q_{k} = \frac{\varGamma (s + k)}{k!\varGamma (s)}\left( {\frac{{R_{c} }}{{s + R_{c} }}} \right)^{k} \left( {\frac{s}{{s + R_{c} }}} \right)^{s},$$

where *s* is known as the “size parameter”.

The size parameter can be chosen freely to vary the amount of overdispersion. Low values of *s* correspond to strong clustering, whereas *q*_*k*_ tends towards the unclustered Poisson distribution as $s \to \infty$.

The same distributions can be used for modelling clustering in the number of infections remaining in the population after MDA. The mean number of infections expected to remain after an MDA of coverage *c* is $I_{r} = \left( { 1 - c} \right)I^{*}$; in the case of homogeneously applied MDA, the number of remaining infections is assumed to take a Poisson distribution around this mean,

$$p_{j} = \frac{{e^{{ - jI_{r} }} }}{j!}I_{r}^{j}.$$

To model clustering, the number of remaining infections is instead assumed to take a negative binomial distribution, with a size parameter controlling the over-dispersion exactly as for the probability generating function for secondary infections,

$$p_{j} = \frac{\varGamma (s + j)}{j!\varGamma (s)}\left( {\frac{{I_{r} }}{{s + I_{r} }}} \right)^{j} \left( {\frac{s}{{s + I_{r} }}} \right)^{s}.$$

The probability of elimination of all infection chains *n* time steps after MDA is

$$z_{n}^{\text {MDA}} = p_0 + \sum_{j=1}^{\infty} {p_j {(z_n)}^j}.$$.

The expected time to extinction starting from *j* infected individuals is derived in [13, 14]:

$${\mathbb{E}}\left[T_{j}^{\text{elim}}\right] = \frac{1}{(1 - \gamma )}\sum\limits_{l = 1}^{N} {\frac{1}{l}\alpha (l)R_{c}^{l - 1} } \mathop \sum \limits_{m = 1}^{{\min(j,l)}} \frac{1}{{\alpha (m)R_{c}^{m - 1} }}.$$

where

$$\alpha \left( l \right) = \frac{N!}{{\left( {N - l} \right)!N^{l} }}.$$

The model could be extended by considering meta-populations comprising multiple connected populations [15], or more straightforwardly by allowing for effects of imported infections. These extensions will always make it harder to eliminate, and present a challenge for maintaining the disease-free state, so the scenarios considered here should be seen as the best case.

The model parameters are listed in Table 1 and R code implementing this model is provided as Additional file 1.

**Table 1:** List of parameters

Symbol	Parameter	Explanation
*I*_*t*_	Number of infectious individuals at time *t*	
N	Total human population	
*β*	Transmission parameter	The expected number of new infections per infectious individual at the next time step
*R*_*c*_	Reproduction number under control	The expected number of secondary infections generated per primary infection at the prevailing coverage of control measures
*γ*	Proportion of population that remains infectious at next time step	Proportion of infectious population that remains infectious at next time step
*q*_*k*_	Probability generating function for secondary infections	Probability a single infection causes *k* infections at the next timestep
*s*	Size parameter	Controls overdispersion of negative binomial distribution
*I*_*r*_	Mean number of infectious individuals remaining post MDA	
*p*_*j*_	Probability generating function for remaining infections	Probability there are *j* infectious individuals remaining post MDA

## Results

**Fig. 1:**
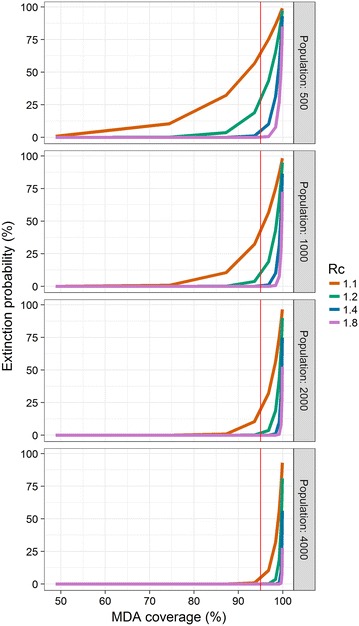
Extinction probability by MDA coverage at various values of *R*_*c*_ and population size. The number of secondary cases and infections post-MDA are assumed to have Poisson distributions. The *vertical red line* indicates 95% MDA coverage

In the absence of clustering of secondary cases or MDA coverage, the probability of achieving elimination with MDA of a given coverage decreases sharply as the population increases and as the reproduction number under control increases. At an MDA coverage of 95% (Fig. 1, marked in red), which is only achievable in interventions with excellent operational characteristics, the probability of elimination is negligible in populations over 1000 or where *R*_*c*_ is greater than 1.2.

**Fig. 2:**
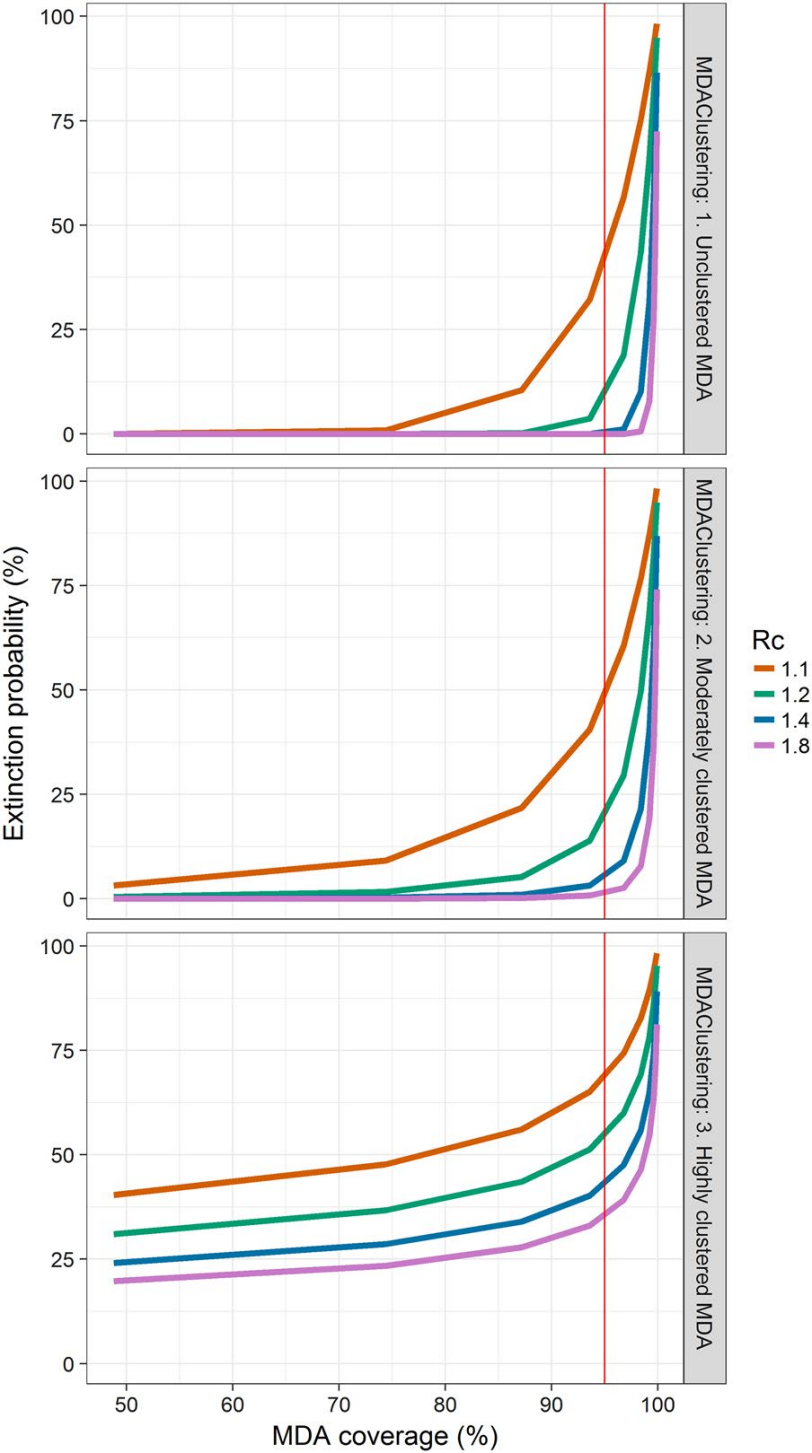
Extinction probability by MDA coverage at various values of *R*_*c*_ and clustering of MDA coverage for a population size of 1000. The number of secondary cases is assumed to have a Poisson distribution, and the number of remaining infections post-MDA takes a negative binomial distribution with size parameter 100 (*top*), 50 (*centre*), 1 (*bottom*). The *vertical red line* indicates 95% MDA coverage

**Fig. 3:**
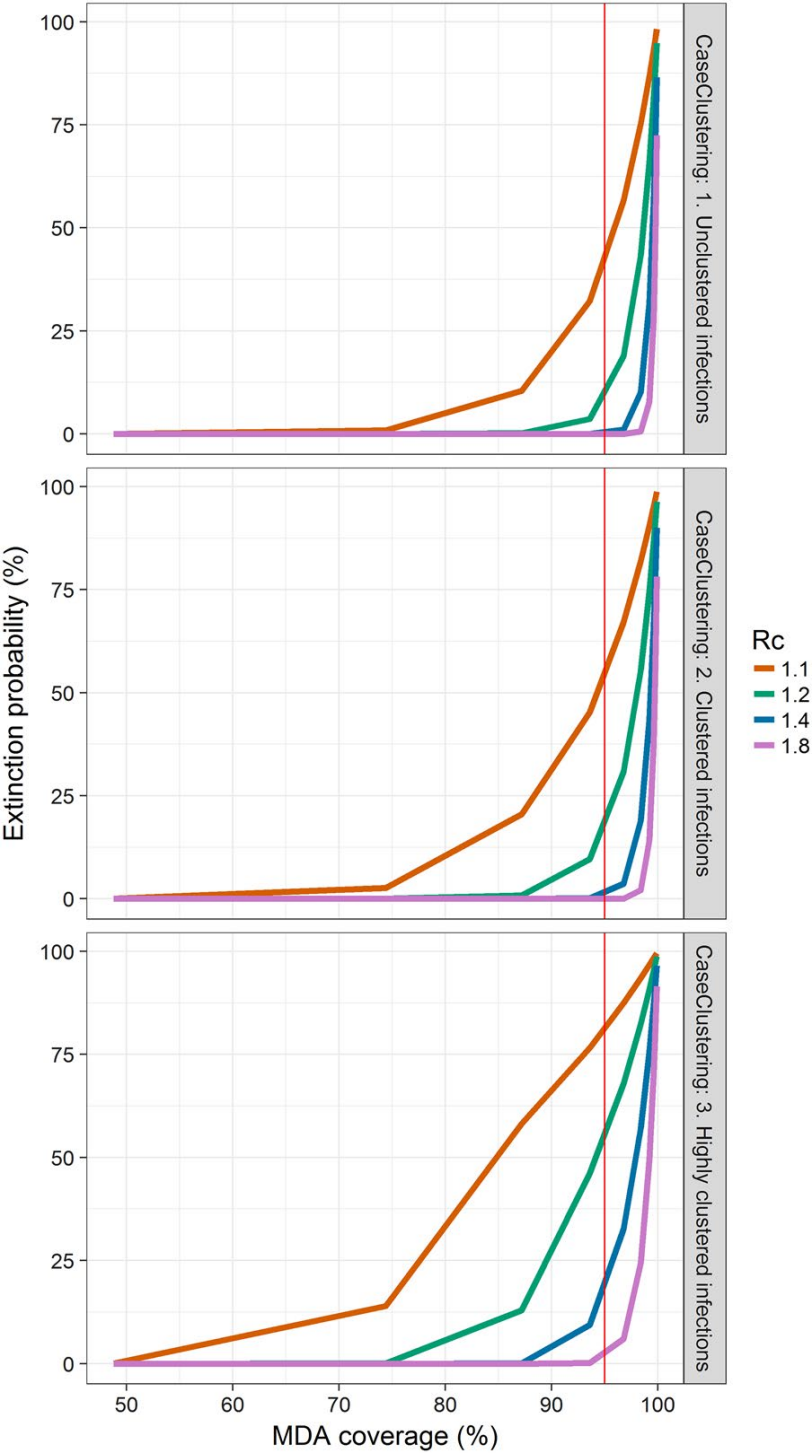
Extinction probability by MDA coverage at various values of *R*_*c*_ and clustering of secondary infections for a population size of 1000. The number of remaining infections post-MDA is assumed to have a Poisson distribution, and the number of secondary infections takes a negative binomial distribution with size parameter 100 (*top*), 50 (*centre*), 1 (*bottom*). The *vertical red line* indicates 95% MDA coverage

In the case where secondary cases result homogeneously from primary cases but MDA coverage is clustered, the model predicts a higher probability of elimination as the clustering in MDA coverage increases (Fig. 2). Clustered infections are expected to increase the probability of elimination when MDA coverage is applied homogenously to the population (Fig. 3), with the effect particularly pronounced at lower values of the reproduction number under control.

**Fig. 4:**
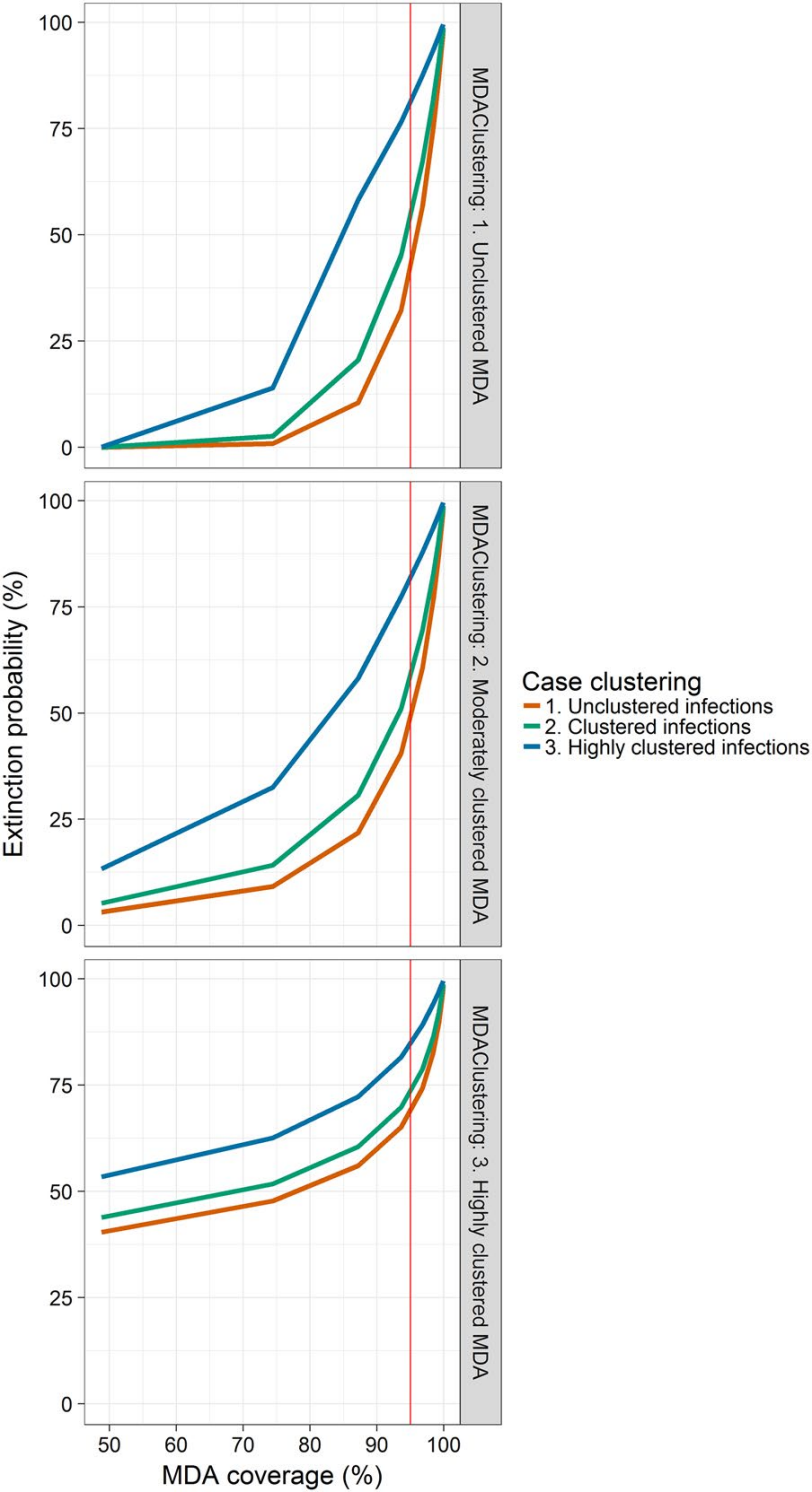
Extinction probability by MDA coverage for clustered MDA coverage and secondary infections for a population size of 1000 and *R*_*c*_ = 1.1. Highly clustered corresponds to size parameter 1, moderately clustered corresponds to size parameter 50, and unclustered corresponds to size parameter 100. The *vertical red line* indicates 95% MDA coverage

Clustering in both secondary cases and MDA coverage produces a substantial increase in the probability of elimination at very low transmission (*R*_*c*_ = 1.1, Fig. 4), and the effect of clustering in MDA treatment has a larger effect than clustering of infections.

**Fig. 5:**
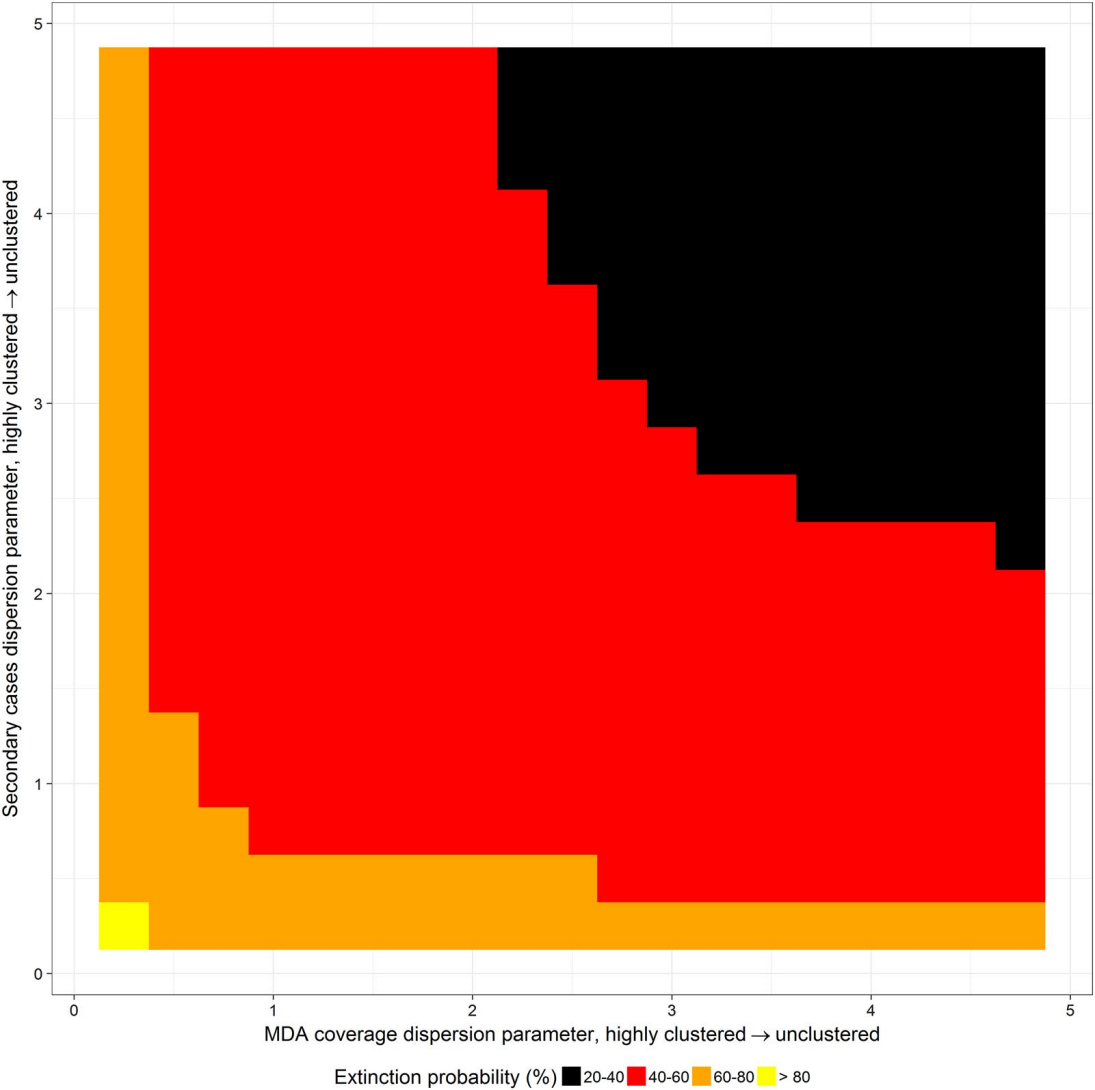
Heatmap showing probability of extinction in idealised setting with 1000 population, 94% MDA coverage and R_c_ = 1.1

In general the probability of elimination from MDA is low. Except for the most idealised of settings, with a small population (N = 1000), very high MDA coverage (94%), very low stable transmission potential (*R*_*c*_ = 1.1), and very high clustering of MDA and secondary infections (Fig. 5), the probability of elimination is low and decreases rapidly as clustering reduces, population size increases or *R*_*c*_ increases.

**Fig. 6:**
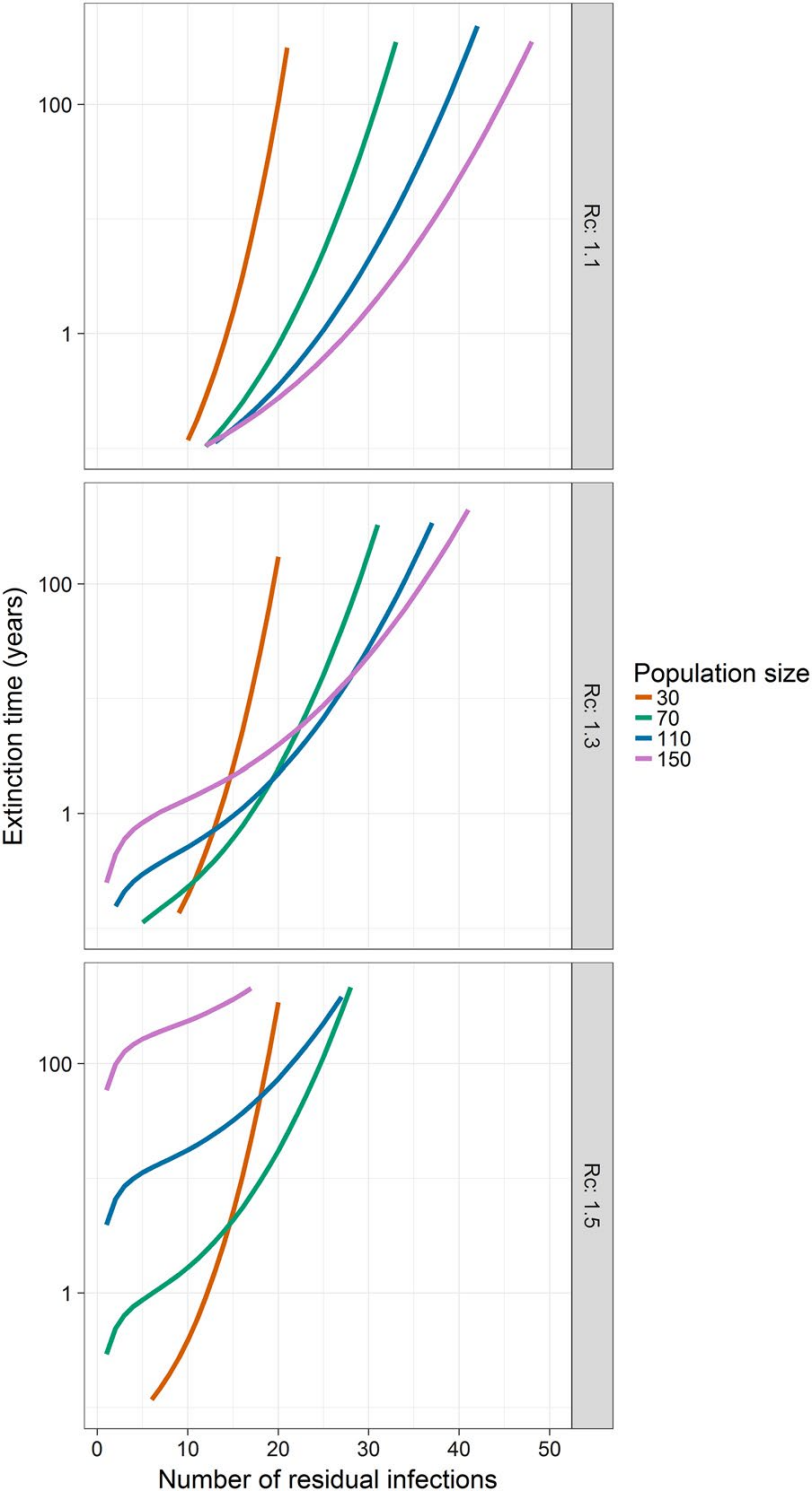
Expected time to extinction by number of residual infections at various population sizes and values of the control reproductive number

So far, this analysis focussed on the probability of achieving elimination after MDA, but the expected time to elimination after an MDA is also of interest. This timeframe is found to increase exponentially as the reproduction number under control increases from 1 (Fig. 6). As MDA coverage increases (the number of residual infections decreases), the range of *R*_*c*_ for which the expected time to elimination remains below 10 years increases modestly, but this range decreases as the population increases. Even in a small population (N = 150) with a very low number of residual infections (15), elimination within 10 years is only expected for $R_c \leq 1.3$. Elimination on operationally relevant timelines (<10 years) at 90% MDA coverage is not expected in populations > 200 unless *R*_*c*_ ≪ 1.1.

## Discussion

The results indicate that mass drug administration (MDA) is only likely to produce a significant probability of local malaria elimination in very limited circumstances. Firstly they suggest that elimination is only likely in situations where *R*_*c*_ is sustained at *R*_*c*_ < 1.2. MDA reduces the infectious reservoir (the proportion of the population infectious to mosquitoes) by the proportion of the population treated. It reduces *R*_*c*_ only because of its temporary prophylactic effect, and once residual drug levels have decayed, *R*_*c*_ returns to the value it would have had without the intervention. For this reason MDA is most effectively deployed in combination with other interventions producing sustained reductions in the force of infection, such as long-lasting insecticide-treated nets, indoor residual spraying or other forms of effective vector control.

Many different patterns of MDA coverage are possible when multiple rounds of treatment are carried out but modelling of this indicates that, at least when rounds are closely-spaced, the most important operational factor determining MDA impact is the proportion of the population who do not receive any MDA treatment at all [16], termed the escape probability. This justifies summarising different patterns of coverage with a single escape probability.

The models show that this escape probability must be very small indeed to achieve a significant probability of local elimination. This not only requires excellent operational logistics in terms of survey and distribution, but also extremely high levels of compliance, community acceptance and adherence [17]. The relevant time-scale over which high coverage must be achieved by MDA is the parasite generation time, and this can account for greater success of MDA with parasites with longer generation times, such as lymphatic filariasis and onchocerciasis. For example, models for lymphatic filariasis in India suggest elimination can be expected if MDA achieves 65% coverage [18] which is comparable to levels achieved in programs [19]. Similar levels of either distribution coverage or compliance would almost certainly fail to eliminate malaria. The longer generation time of *P. vivax* could account for why many relatively successful MDA programs have been against *P. vivax* (using drug regimens that are effective against hypnozoites) rather than *P. falciparum* [17]. In most scenarios modelled, effective cure rates of >95% would be required to have a non-negligible probability of successful elimination.

In deterministic population models of infection dynamics, extinction occurs either when *R*_*c*_ is sustained below 1, or the infectious reservoir is reduced to zero. Neither condition is likely to be satisfied by a time-limited MDA program. In particular, reduction of the infectious reservoir to zero requires complete (100%) coverage. However in a stochastic model, as in the real world, the infectious reservoir may reach zero as a result of chance events. In particular, if the population of hosts or parasites is small, as was the case in particular in Aneityum, Vanuatu [3], a pulsed intervention might be effective. However other MDA programs, notably that of the Garki project [20], have failed to achieve even local elimination [21]. The results suggest that the maximum population size at which it may be possible to take advantage of stochastic fluctuations to achieve elimination is of the order 1000, given ideal circumstances. As the transmission potential and escape probability increase, this maximal population size decreases yet further. MDA may have a role to play in achieving local elimination in small, isolated populations, for example on small islands or in residual pockets of transmission inside a larger area where elimination has been otherwise achieved. Small populations may also be more favourable to achieving the required population coverage and compliance rates.

As a pulsed intervention, MDA is expected to achieve elimination very soon after implementation, or not at all. However in the stochastic SIS model the infection will eventually go extinct anyway, but this takes an extremely long time with realistic population sizes and parameterisations. The expected time to elimination increases exponentially in *R*_*c*_ and for a population of 150, it is at least 10 years for *R*_*c*_ > 1.3.

An important factor that not analysed here is temporal variation in transmission intensity, especially seasonality. MDA has a greater impact and is more likely to achieve elimination if carried out at a time when the parasite reservoir and the potential for reinfection is small [21–24].

Clustering of secondary infections and of MDA distribution both contribute positively to the potential probability of success. In low prevalence settings cases are expected to be more spatially clustered than at higher prevalence, meaning that clustering should improve the chances of elimination those places where elimination is most likely to be feasible. Clustering of MDA coverage would most likely take the form of administration of drugs to entire households, either through door-to-door campaigns or centralised provision of sufficient tablets of a single-dose cure to one household member. The success of this approach lies in the over-representation of cases in households with an existing case, so that at sufficiently high coverage and low prevalence any missed households are more likely to be entirely disease free.

The MDA is modelled here as a single intervention yielding a single probability of success. Even if this probability is small, it is tempting to consider repeating the MDA multiple times to produce a higher overall probability of success (for example, naively modelling each of n rounds of MDA as having an independent chance of success *p* yields a probability of at least one success of 1 − (1 − *p*)^*n*^. In practice rounds of MDA are likely to be highly correlated in coverage, yielding strongly diminishing returns particularly if there remains a constant fraction of the population who are never successfully treated. Repeated MDAs could also have detrimental effects on parasite resistance to the drug used and on community compliance rates, either of which could impact negatively on the effective cure rate at subsequent rounds.

## Conclusions

MDA is expected to produce a significant probability of elimination only in areas of small populations with low *R*_*c*_ where almost 100% of the population can be successfully treated. In other circumstances, transient reductions in prevalence are expected to be temporary. Stochastic fluctuations caused by noise in the number of infections is only significant on timescales of interest in populations smaller than about 1000 people. Treatments of entire households may increase the probability of successful elimination at low prevalence.

## Additional file

**Additional file 1.** R code for calculating extinction probabilities.
